# Protective effects of glaucocalyxin A on the airway of asthmatic mice

**DOI:** 10.1515/med-2022-0513

**Published:** 2022-07-07

**Authors:** Si Chen, Ying Piao, Yilan Song, Zhiguang Wang, Jingzhi Jiang, Yihua Piao, Li Li, Chang Xu, Liangchang Li, Yongxue Chi, Guihua Jin, Guanghai Yan

**Affiliations:** Department of Pediatrics, Affiliated Hospital of Yanbian University, Yanji 133099, Jilin, P. R. China; Department of Neonatology, Children’s Hospital of Changchun, Changchun 130061, Jilin, P. R. China; Jilin Key Laboratory for Immune and Targeting Research on Common Allergic Diseases, Yanbian University, Yanji 133000, Jilin, P. R. China; Department of Emergency, Yanbian University Hospital, Yanji 133000, Jilin, P. R. China; Department of Anatomy, Histology and Embryology, Yanbian University Medical College, Yanji 133002, Jilin, P. R. China; Department of Pediatrics, Affiliated Hospital of Yanbian University, No. 1327, Juzi Street, Yanji 133099, Jilin, P. R. China; Department of Immunology and Pathogenic Biology, Yanbian University Medical College, No. 977, Gongyuan Road, Yanji 133002, Jilin, P. R. China; Department of Respiratory Medicine, Affiliated Hospital of Yanbian University, Yanji 133000, Jilin, P. R. China; Department of Intensive Care Unit, Affiliated Hospital of Yanbian University, Yanji 133000, Jilin, P. R. China; Department of Anatomy, Histology and Embryology, Yanbian University Medical College, No. 977, Gongyuan Road, Yanji 133002, Jilin, P. R. China

**Keywords:** glaucocalyxin A, asthma, airway, TLR4/NF-κB signaling pathway, inflammatory responses

## Abstract

The aim of this study is to investigate the protective effects of glaucocalyxin A (GLA) on airways in mouse models of asthma, concerning the inflammatory mediators, Th1/Th2 subgroup imbalance, and Toll-like receptor 4 (TLR4)/NF-κB signaling pathway. Hematoxylin and eosin/periodic acid–Schiff staining was used to observe the pathological changes in lung tissues. Inflammatory cytokine contents in the bronchoalveolar lavage fluid were detected by enzyme-linked immunosorbent assay. Protein expression levels were detected with Western blot, immunohistochemistry, and immunofluorescence. *In vivo* studies showed that, in ovalbumin (OVA)-induced asthmatic mouse models, the GLA treatments reduced the airway hyperresponsiveness and the secretion of inflammatory cells, declined the proliferation of goblet cells, decreased the levels of IL-4, IL-5, and IL-13, and increased the contents of interferon-γ and IL-12. Moreover, GLA inhibited the protein expression levels of TLR4, MyD88, TRAF6, and NF-κB in OVA-induced asthmatic mouse models. Further *in vitro* studies showed that GLA inhibited the expression of NF-κB, p-IκBα, tumor necrosis factor-α, IL-6, and IL-1β and blocked the nuclear transfer of NF-κB in lipopolysaccharide-stimulated RAW264.7 macrophages. Conclusively, GLA can inhibit the inflammatory responses in OVA-induced asthmatic mice and inhibit the release of inflammatory factors in LPS-induced RAW264.7 macrophages, which may be related to the inhibition of TLR4/NF-κB signaling pathway.

## 1 Introduction

Bronchial asthma is a common chronic disease of the respiratory system, mainly mediated by the effector cells (such as eosinophils). Bronchial asthma is always accompanied by the accumulation of various inflammatory factors, which might lead to bronchial lumen stenosis and pathological changes in the lung tissue. Nowadays, increasing environmental pollution, involuntarily external contact, and instantaneous emotional changes would represent the inducing factors for bronchial asthma, bringing huge physical and mental suffering to patients [1,2].

For the patients with bronchial asthma, the respiratory tract is highly sensitive to the external factors. On the other hand, in the human bodies, the differentiation of the Th0 cells into the Th2 cells, together with the excessive formation of IgE and inflammatory mediators, would lead to hypersensitivity and inflammatory-waterfall effects [3,4]. Moreover, the Th2 cells would infiltrate into the airway tract and lung tissue, which can stimulate the airway mucosa and epithelial smooth muscle to produce more inflammatory factors, thus aggravating the inflammatory infiltration and ultimately leading to asthma [5,6]. In recent years, the Toll-like receptor 4 (TLR4)/NF-κB signaling pathway and the airway inflammation have attracted much attention in asthma research.

TLR4 adaptively activates the immune-related genes in the immune responses, which identifies various pathogen-associated molecular patterns (PAMPs, such as lipopolysaccharide [LPS]), and therefore represents the primary barrier against allergic diseases. After activation, the inflammatory mediators increase and the nonspecific immunity predominates, causing immune imbalance and leading to asthma. On the other hand, in an activated state, NF-κB would be transferred into the nucleus and bind to a specific sequence of DNA, therefore involving in the regulation of cellular immune responses [7,8]. In a previous study, the asthmatic mice were divided into two groups. In the first group with the presence of the p50 gene and c-Rel subunit of NF-κB, the asthmatic symptoms were obvious after challenging; while in the second group with NF-κB p50 gene knocked out and the c-Rel subunit silencing, the mice had no obvious asthmatic symptoms (no high sensitivity) after challenging [9]. These findings suggest that the occurrence of asthma airway inflammation cascade may be related to the expression of NF-κB.

The first defense barrier of the body’s immune response against the invading pathogens is achieved by the pattern recognition receptors on the antigen presenting cells, in combination with the PAMPs. When stimulated by the allergen (such as the infection or inflammatory mediators), TLR4 would bind to PAMPs to transfer the signal into the cells, by acting with the linker molecule MyD88. Thereafter, NF-κB migrates into the nucleus to couple with the DNA-specific sequence fragments [10,11], including the tumor necrosis factor (TNF)-α, cyclooxygenase 2, IL-4, and IL-5. Activation of NF-κB initiates the expression of a variety of inflammatory mediators, thus inducing the inflammatory reactions [12,13]. However, the signaling regulation is somewhat restrictive. If the pathway is continuously activated, it will lead to the overexpression of related inflammatory factors, further resulting in the inflammatory reaction and even autoimmune diseases. On the contrary, under the weak pathway signaling condition, inflammatory factors are insufficiently synthesized, and the immune system is unable to form a barrier to protect the body. It has been shown that the activated signaling pathway would block the downstream processes, which makes NF-κB fail to bind to DNA or promote DNA self-repairing, thereby regulating the body’s immune response [14].

Glaucocalyxin A (GLA) is a diterpenoid compound extracted from cymbidium calyx [15,16]. GLA has the effects of clearing heat and detoxification, promoting blood circulation, and removing blood stasis in traditional Chinese medicine [17]. In recent years, the anti-inflammatory, immune protection, and anti-tumor effects of GLA have also been demonstrated. For example, GLA can regulate the cellular inflammatory responses by inhibiting the NF-κB signaling pathway. Moreover, GLA can attenuate allergic responses and inhibit mast cell degranulation through p38MAPK/NrF2/HO-1 and HMGB1/TLR4/NF-κB pathways [18]. In addition, it is shown that GLA could reverse the lung fibrosis in mice [19]. However, there are few studies concerning the role and mechanism of GLA in asthma.

In this study, to investigate whether GLA could inhibit the occurrence of bronchial asthma, a mouse model of asthma was established with ovalbumin (OVA). The inhibitory effects of GLA on the TLR4/NF-κB signaling pathway in the asthmatic mice were analyzed.

## 2 Materials and methods

### 2.1 Study animals

A total of 40 female BALB/C mice (6–8 weeks old; weighing 18–20 g) were purchased from the Yanbian University Animal Experimental Center. These animals were kept in a 12/12 h light and dark cycle, with free access to standard food and drinking water. These mice were randomly divided into the following five groups (*n* = 8 each group): the saline (SAL), OVA-sensitized, dexamethasone (1 mg/kg) (Dex), and 10 and 20 mg/kg GLA groups. Animal experimental procedures were approved by the Yanbian University Medical Ethics Committee.

### 2.2 Cell culture and treatment

**Figure 1:**
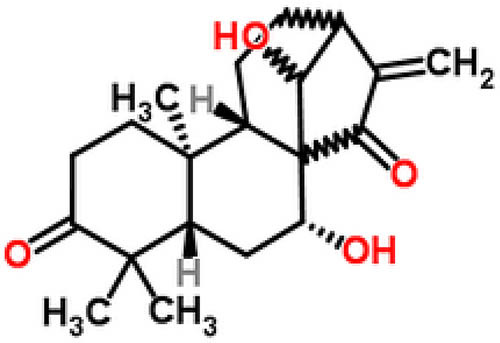
Chemical structure of GLA (C20H28O4).

For the *in vitro* model, RAW264.7 cells were cultured with dulbecco’s modified eagle medium containing 10% fetal calf serum, 4 mmol/L glutamine, and 1% penicillin-streptomycin (Gibco) under 5% CO_2_ at 37°C. For treatment, cells were first stimulated with LPS (100 ng/mL) for 24 h and then treated with different concentrations of GLA (0, 0.1, 1, and 5 μM) for another 24 h as previously reported [20]. GLA was purchased from Shanghai Yuanye Biotechnology (Cat. No., B20698; China). The purity of GLA was over 99%, which was white crystal, with the melting point of 513.4°C. The chemical structure of GLA is shown in Figure 1.

### 2.3 Chronic allergic asthma model establishment

**Figure 2:**
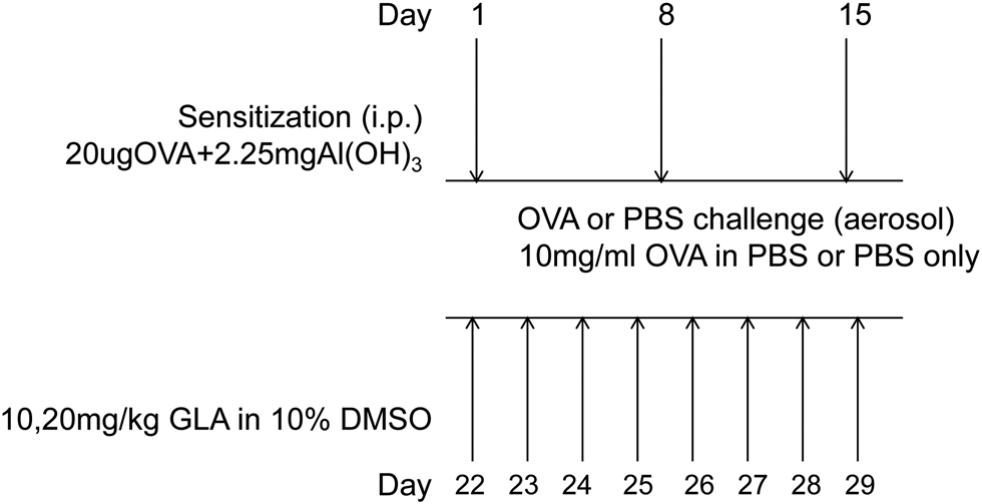
Flow chart for the establishment of mouse asthma model (*N* = 8).

Animals were adaptively reared for 1 week. Then, on days 1, 8, and 15, except for the SAL group, the mice from other groups were sensitized with 20 μL of OVA (Sigma-Aldrich Co, USA) + 2.25 mg aluminum hydroxide adjuvant (Al(OH)_3_, Sigma-Aldrich Co, USA) in 200 μL intraperitoneal injection (Figure 2). On day 22, the mice were challenged with 3% OVA, through aerosol infusion for 1/2 h, for 7 consecutive days. At 1 h before the fifth day of nebulization, 1 mg/kg Dex and 10 mg/kg (or 20 mg/kg) GLA were injected into the peritoneal cavity, for 3 consecutive days. The GLA dose was determined based on the previous study [19]. After the challenge, the mice showed typical symptoms of asthma, including shortness of breath, snoring, irritability, and fear.

### 2.4 Bronchoalveolar lavage fluid (BALF) and lung sample preparation

At 24 h after the last nebulization, the mice were anesthetized by ether. After disinfection, a longitudinal cut was made on the neck skin, and the tissues were separated until the trachea was exposed. A small opening was cut, and the catheter was inserted and tied with surgical thread. The lungs were repeatedly flushed three times with 0.5 mL of cold SAL, and the volume of BALF was not less than 0.4 mL. BALF was centrifuged at 500×*g* at 4°C for 5 min, and the supernatant was kept at −80°C. Remaining cell suspensions were used to prepare the cyto-smears. Moreover, the lung was collected.

### 2.5 Airway hyperresponsiveness assessment

All animals underwent bronchial stimulation test at 24 h after inhalation of OVA. The mice were anesthetized by the intraperitoneal injection of 4% pentobarbital at the dose of 60 mg/kg body weight. After the pain reflex disappeared, the mice were placed supine in the plethysmography box of experimental Animal Lung Function Detection and Analysis System (AniRes2005, Beijing Bellambo Technology Co., Ltd.), and the skin was cut open to separate the subcutaneous tissues. The exposed trachea was connected to an animal ventilator and an endotracheal tube was inserted. The external jugular vein was dissociated and punctured, the needle handle was fixed, and the body tracing box was closed. Methacholine (2.5, 5.0, 10, 25, and 50 g/L) was injected intravenously (0.2 mL each time) through the external jugular vein. The bronchopulmonary resistances were expressed as enhanced pauses (Penh). The results were expressed as the percentage increase in Penh over the baseline, where the baseline Penh was expressed as 100%.

### 2.6 BALF and inflammatory cell counts

The BALF was centrifuged at 50×*g* for 10 min, and the precipitate was resuspended in 500 μL of normal SAL. To count the inflammatory cells, a total of 300 cells were enumerated from three different random sites of each smear under 1,000× magnification. The number of macrophages, lymphocytes, eosinophils, and neutrophils and the total number of cells were determined.

### 2.7 Histological staining

The lung tissue was fixed in formaldehyde, dehydrated, paraffin-embedded, and cut into 4 μm sections. Sections were stained with hematoxylin and eosin (H&E) to assess the lung inflammation. More than five high-power fields were randomly selected from each group to observe the histopathological changes. The severity of peribronchial inflammation was graded semiquantitatively using the following criteria: 0 points, normal; 1 point, few cells; 2 points, a ring of inflammatory cells of 1 layer; 3 points, a ring of inflammatory cells of 2–4 layers; and 4 points, a ring of inflammatory cells of 4 layers. Periodic acid–Schiff (PAS) staining was also performed to observe goblet cell proliferation and mucus production. The number of PAS-positive cells (goblet cells) and the number of epithelial cells were counted. According to previous studies [21,22], the percentage of PAS-positive goblet cells was calculated as the ratio of the number of goblet cells to the total number of epithelial cells in the bronchus

### 2.8 Enzyme-linked immunosorbent assay (ELISA)

The contents of IL-4, IL-5, IL-12, IL-13, and interferon-γ (IFN-γ) in BALF were determined with ELISA kits (R&D systems, USA), according to the manufacturer’s instructions. Briefly, the biotin antibody, enzyme-conjugated working solution, and substrate solution were sequentially added to the plate, and the plate was washed between the tests for different samples. Optical density was measured by a microplate reader, and the concentrations were calculated based on the standard curves.

For the measurement of TNF-α, IL-6, and IL-1β produced by GLA-treated macrophages, the RAW264.7 cells were stimulated with LPS, in the presence or absence of GLA (0.1, 1, and 5 μM, respectively), at 37°C for 24 h. The culture supernatant was harvested and prepared. The cytokine concentrations were determined with the kits as described above.

### 2.9 Immunohistochemistry

The expression levels of TLR4 in the lung samples were detected with immunohistochemistry. The sections were placed in a 60°C dryer, which were then subjected to the treatment of xylene de-paraffin, dehydration with gradient ethanol, and citrate antigen repairing. The sections were incubated with the anti-TLR4 (#20379, 1:1,000; Beijing Biosynthesis Biotechnology Co., Ltd, China) at 4°C overnight. Then, the section was incubated with anti-mouse secondary antibody (# 5151, 1:1,500; Cell Signaling Technology, USA) at room temperature for 1 h. Color development was performed with the diaminobenzidine method. After hematoxylin counterstaining, dehydrating, and washing, the sections were observed under microscope. Five different fields of view were randomly selected and observed at 400× magnification.

### 2.10 Western blot analysis

Tissues and cells were lysed for protein extraction. Protein concentrations were determined with the bicinchoninic acid methods. The protein samples were separated by 10% sodium dodecyl sulfate-polyacrylamide gel electrophoresis and then electronically transferred onto the membrane. The membranes were blocked with the skimmed milk at room temperature for 1 h and then washed before antibody incubation. For the detection of protein levels in tissues, the membrane was incubated with primary antibodies of TLR4, NF-κB (#8242, 1:1,000), MyD88 (#50010, 1:1,000), IκB-α (#4814, 1:1,000), TRAF6 (#8028, 1:1,000), and β-actin (#3700, 1:1,500) at 4°C overnight. For the detection of protein levels in cells, the membrane was incubated with primary antibodies of NF-κB (#8242, 1:1,000), p-IκBα (#9246, 1:1,000), IκB-α (#4814, 1:1,000), and glyceraldehyde-3-phosphate dehydrogenase (GAPDH) (#97166, 1:1,000) at 4°C overnight. After washing, the membrane was incubated with anti-mouse secondary antibody (#5151, 1:1,500) at room temperature for 1 h. The aforementioned antibodies are all from Cell Signaling Technology. Color development was performed with the electrochemiluminescence method. The images of the protein bands were obtained and analyzed by ImageJ software. β-Actin and GAPDH were used as internal reference.

### 2.11 Immunofluorescence

The expression level of NF-κBp65 was detected with immunofluorescence. The lung tissue section was degraded with ethanol gradients, repaired with citrate antigen. For the detection of NF-κBp65 in the RAW264.7 cells, treated cells were fixed with 4% paraformaldehyde. The prepared lung sections and cells were permeabilized with 0.2% Triton X-100. After blocking with 10% goat serum, the samples were incubated with anti-NF-κBp65 (#8242, 1:1,000; Cell Signaling Technology) at 4°C overnight. Then, the samples were incubated with Donkey Anti-Rabbit IgG H&L (Alexa Fluor® 488) (ab150073) for 2 h at room temperature. The immunofluorescence was observed with laser confocal microscopy (Cytaion 5, Beckman, USA).

### 2.12 3-(4,5-dimethylthiazol-2-yl)-2,5-diphenyltetrazolium bromide (MTT) assay

The cells in the logarithmic growth phase were subjected to the viability assessment with the MTT assay. Cells were seeded on the 96-well plate and treated with different concentrations of GLA (0, 0.1, 1, and 5 μM). When the confluence reached 80%, each well was incubated with 5 mg/mL of MTT for 4 h. Then, the supernatant was discarded, and 150 μL of dimethyl sulfoxide (#12611; Cell Signaling Technology) was added. The optical density at 570 nm was measured with a microplate reader (Bio-Rad Labs, Sunnyvale, CA, USA), and cell viability was calculated accordingly.

### 2.13 Statistical analysis

Data were expressed as mean ± SD. SPSS 22.0 software (SPSS Inc., Chicago, IL, USA) was used for statistical analysis. Data distribution was analyzed. The *t*-test was used for the pairwise group comparison of data with normal distribution. Multiple comparisons among groups were performed using analysis of variance (ANOVA) (for data with normal distribution) or Wilcoxon’s rank-sum test (for data with non-normal distribution). The experiment was repeated three times. *P* < 0.05 was considered statistically significant.

## 3 Results

### 3.1 Effects of GLA on airway hyperresponsiveness

**Figure 3:**
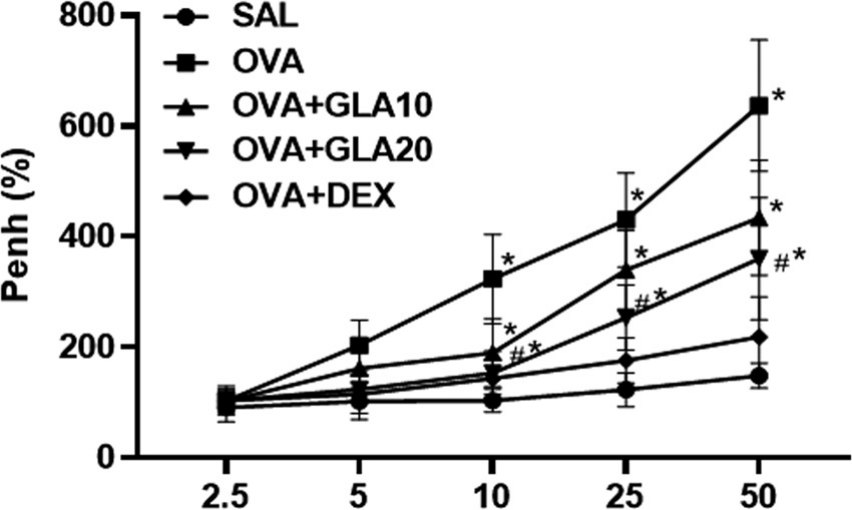
Effects of GLA on the airway hyperresponsiveness (AHR) to methacholine. The baseline Penh of the SAL-treated control group is considered 100%. The percentage increase in Penh over the baseline is shown. *N* = 3; ^#^ *P* < 0. 01 vs the SAL group; ^*^ *P* < 0.05 vs the OVA group; analyzed by ANOVA.

As the concentration of methylacetylcholine increased exponentially, inspiratory resistance was increased in both the OVA group and the SAL control group. When the concentration reached more than 10 g/L, there was a significant difference between the OVA + GLA group and the OVA group (*P* < 0. 01), as shown in Figure 3. This suggests that GLA could inhibit airway hyperresponsiveness.

### 3.2 Effects of GLA on inflammatory cells

**Figure 4:**
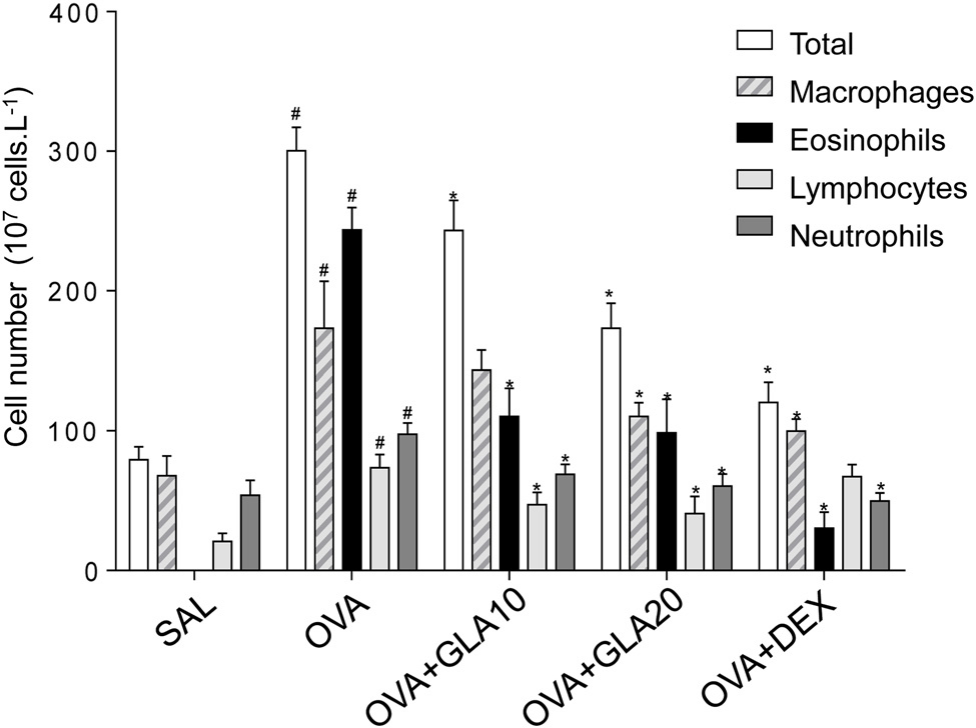
Effects of GLA on inflammatory cell counts in BALF. The numbers of total inflammatory cells, macrophages, lymphocytes, eosinophils, and neutrophils were counted in the BALF in the SAL, OVA-sensitized, Dex (1 mg/kg), and 10 and 20 mg/kg GLA groups. Compared with the SAL group, ^#^ *P* < 0.05; and compared with the OVA-sensitized group, ^*^ *P* < 0.05. *N* = 3; analyzed by ANOVA.

The total cells and inflammatory cells in the BALF were counted. Our results showed that, compared with the SAL group, the counts of the total cells, macrophages, lymphocytes, eosinophils, and neutrophils in the OVA-sensitized group were significantly higher, which were decreased in the GLA groups with different doses, with statistical significance for the high-dose GLA group (*P* < 0.05) (Figure 4).

### 3.3 Effects of GLA on lung histopathology

**Figure 5:**
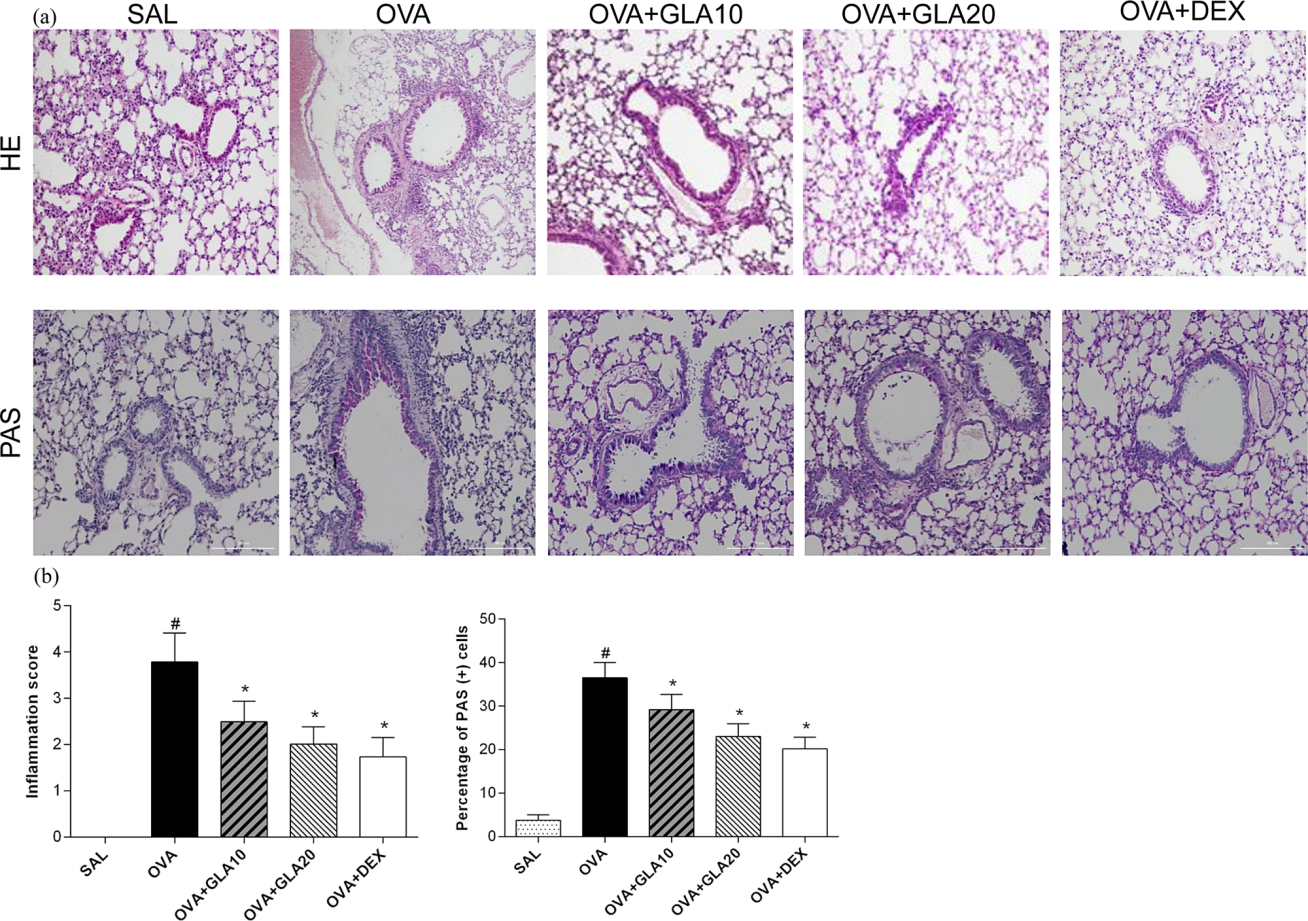
Effects of GLA on histological changes in lung tissues of OVA-sensitized asthmatic mice. (a) Lung sections were stained with H&E (200×) to measure the inflammatory cell infiltration and stained with PAS (400×) to detect the goblet cell hyperplasia, in the SAL, OVA-sensitized, Dex (1 mg/kg), and 10 and 20 mg/kg GLA groups. (b) Inflammation score and percentage of PAS (+) cells. ^#^ *P* < 0.05 vs the control mice, ^*^ *P* < 0.05 vs the OVA mice. *N* = 3; analyzed by ANOVA.

Next, the effects of GLA on the pathological features of lung tissue were detected by the H&E and PAS staining. Our results from the H&E staining showed that, compared with the SAL group, a large amount of secretion around the airway wall was observed, to cause mucus plug accumulation, in the OVA-sensitized group (Figure 5a). However, after the GLA treatment, inflammatory secretion around the airway was reduced, in a dose-dependent manner. Moreover, the pathological changes of lung tissue in the Dex group were lighter, with no mucus plug formation. Statistically, OVA treatment induced significantly increased inflammation score than control, while GLA and Dex treatment significantly decreased inflammation score than OVA (*P* < 0.05) (Figure 5b). These results suggest that GLA can reverse the pathological damages of lung tissue in asthmatic mice. On the other hand, our results from the PAS staining showed the adhesion of inflammatory cells and the invasion of goblet cells in the SAL group (Figure 5a). However, in the OVA-sensitized group, a large number of inflammatory cells were observed around the airway wall, with the excessive invasion of goblet cells. Moreover, these pathological damages were improved in the GLA groups of different doses, in which the numbers of inflammatory cells and goblet cells were reduced. Statistically, compared to control, OVA treatment significantly increased the percentage of PAS(+) cells. However, compared with OVA, GLA and Dex treatment significantly decreased the percentage of PAS(+) cells (*P* < 0.05) (Figure 5b). These results suggest that GLA can inhibit the formation of mucus plugs around the airway wall and the proliferation of goblet cells.

### 3.4 Effects of GLA on cytokine expression levels

**Figure 6:**
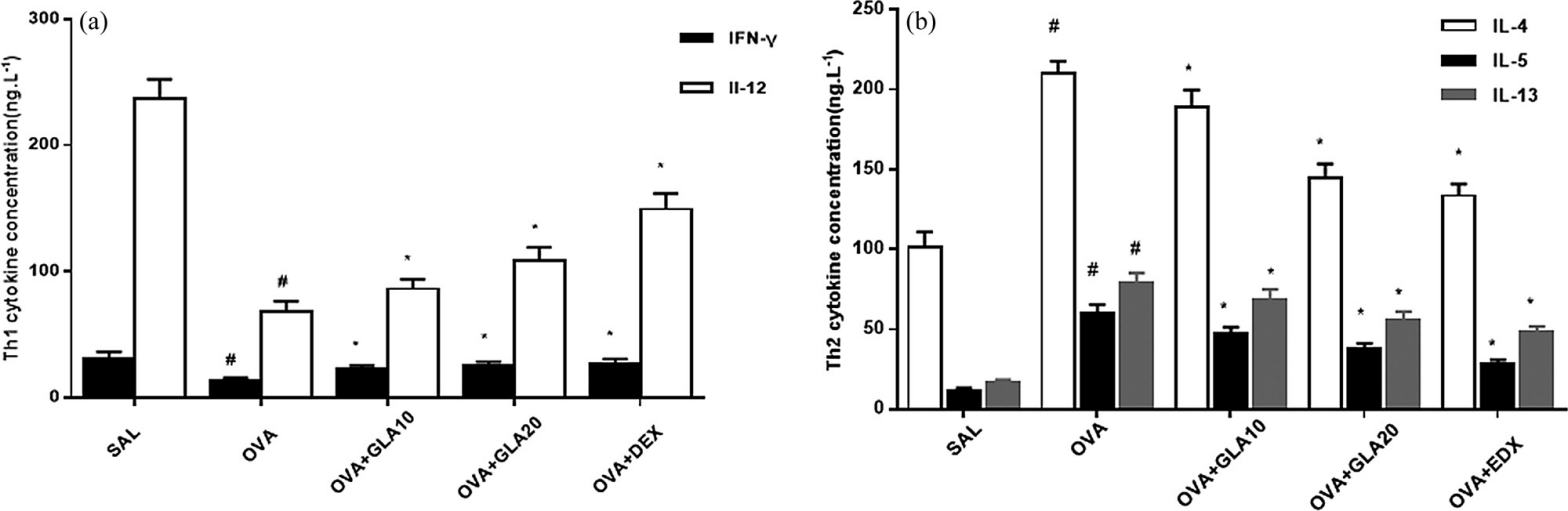
Effects of GLA on cytokine expression levels. (a) Levels of IL-4, IL-5, and IL-13 in the BALF were analyzed using ELISA. (b) Levels of IL-12 and IFN-γ in the BALF were analyzed using ELISA. Compared with the SAL group, ^#^ *P* < 0.05; and compared with the OVA-sensitized group, ^*^ *P* < 0.05. *N* = 3; analyzed by ANOVA.

Subsequently, the expression levels of IL-4, IL-5, IL-13, IL-12, and IFN-γ were determined by ELISA. Our results showed that, compared with the SAL group, the expression levels of IL-12 and IFN-γ were significantly decreased (Figure 6a), while the expression levels of IL-4, IL-5, and IL-13 were significantly increased (Figure 6b) in the OVA-sensitized group. However, after the treatment of GLA at appropriate concentrations, the expression levels of IL-12 and IFN-γ were significantly increased (*P* < 0.05), while the expression levels of IL-4, IL-5, and IL-13 were significantly decreased (*P* < 0.05). These results suggest that GLA may have regulatory effects on related cytokine expression levels during the asthma attack.

### 3.5 Effects of GLA on TLR4 protein expression

**Figure 7:**
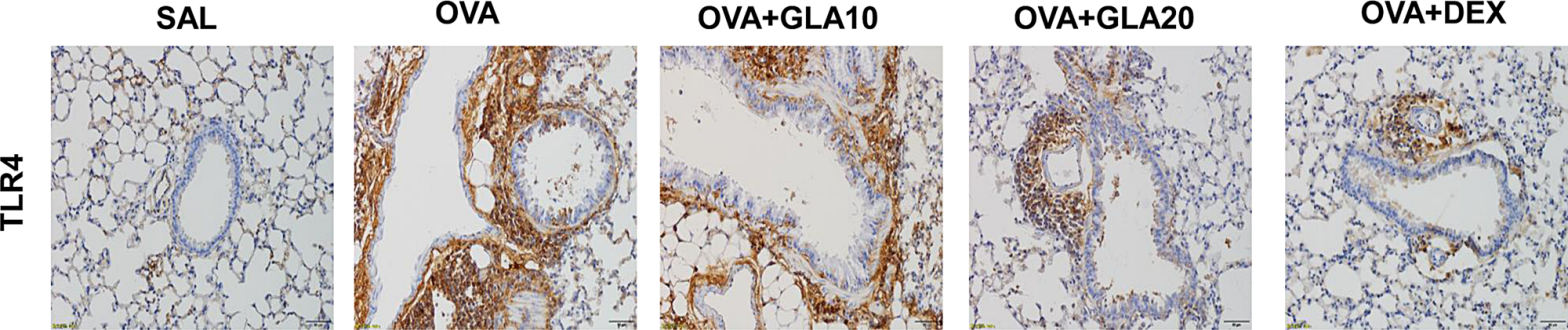
Immunohistochemical staining of lung tissues in asthmatic mice. Representative histological images of the lung tissues in the SAL, OVA-sensitized, Dex (1 mg/kg), and 10 and 20 mg/kg GLA groups (200×).

Immunohistochemistry was performed to observe the expression levels of TLR4 in the lung tissue. Our results showed that there were no pathological damages in the lung tissue in the SAL group, and the TLR4 expression was rarely seen. However, in the OVA-sensitized group, a large amount of TLR4 protein expression was observed (staining dark brown-yellow). Moreover, after the GLA treatment, the expression levels of TLR4 around the bronchi were reduced, which was more obvious in the lung tissue for the high-dose GLA group (Figure 7).

### 3.6 Effects of TLR4, NF-κBp65, TRAF-6, IκB-α, and MyD88 expression levels

**Figure 8:**
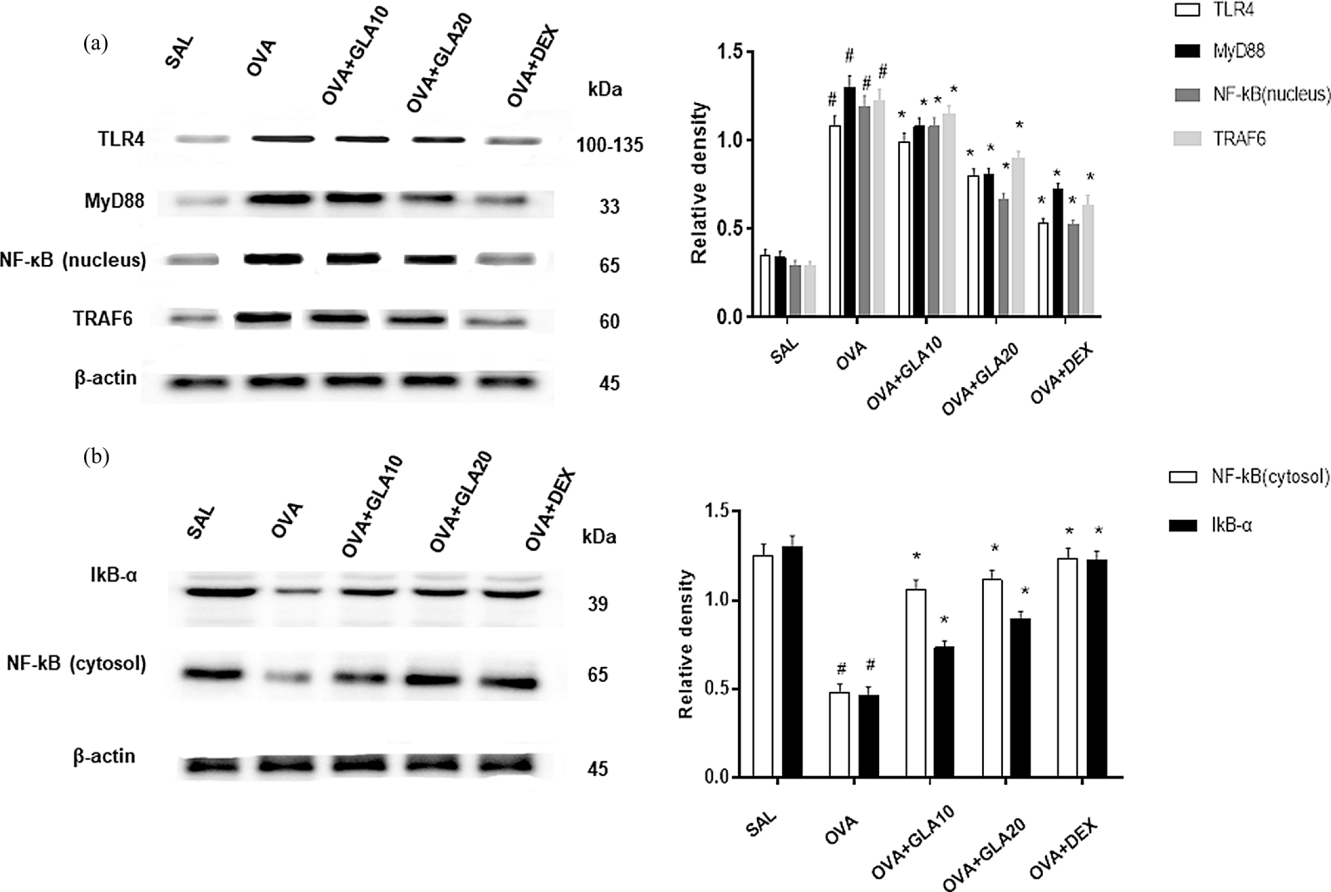
Effects of GLA on TLR4, NF-κBp65, TRAF-6, IκB-α, and MyD88 expression and NF-κB translocation. (a) The protein expression levels of TLR4, NF-κBp65 (nucleus), TRAF-6, IκB-α, and MyD88 expression, as well as the NF-κB translocation, in the SAL, OVA-sensitized, Dex (1 mg/kg), and 10 and 20 mg/kg GLA groups, were detected with Western blot analysis. (b) The protein expression levels of NF-κBp65 (cytosol) and IκB-α expression, as well as the NF-κB translocation, in the SAL, OVA-sensitized, Dex (1 mg/kg), and 10 and 20 mg/kg GLA groups, were detected with Western blot analysis. Compared with the SAL group, ^#^ *P* < 0.05; and compared with the OVA-sensitized group, ^*^ *P* < 0.05. *N* = 3; analyzed by ANOVA.

The TLR4/NF-κB signaling pathway plays an important role in the mechanism underlying the asthmatic inflammatory response. Therefore, the effects of GLA on the expression levels of related proteins (TLR4, NF-κB, IκB-α, TRAF6, and MyD88) in the signaling pathway were investigated by Western blot analysis. As shown in Figure 8, our results showed that the expression levels of TLR4, MyD88, NF-κB (nucleus), and TRAF6 were significantly increased, while the expression levels of NF-κB (cytosol) and IκB-α were significantly decreased, in the OVA-sensitized group. In the GLA groups with different doses, the expression levels of TLR4, MyD88, NF-κB (nucleus), and TRAF6 were significantly decreased (*P* < 0.05), while the expression levels of NF-κB (cytosol) and IκB-α were significantly increased (*P* < 0.05), in a dose-dependent manner. These results suggest that GLA may inhibit the nuclear transfer of NF-κB by interfering with the activation of NF-κB.

### 3.7 Effects of GLA on NF-κBp65 expression levels in the lung tissue

**Figure 9:**
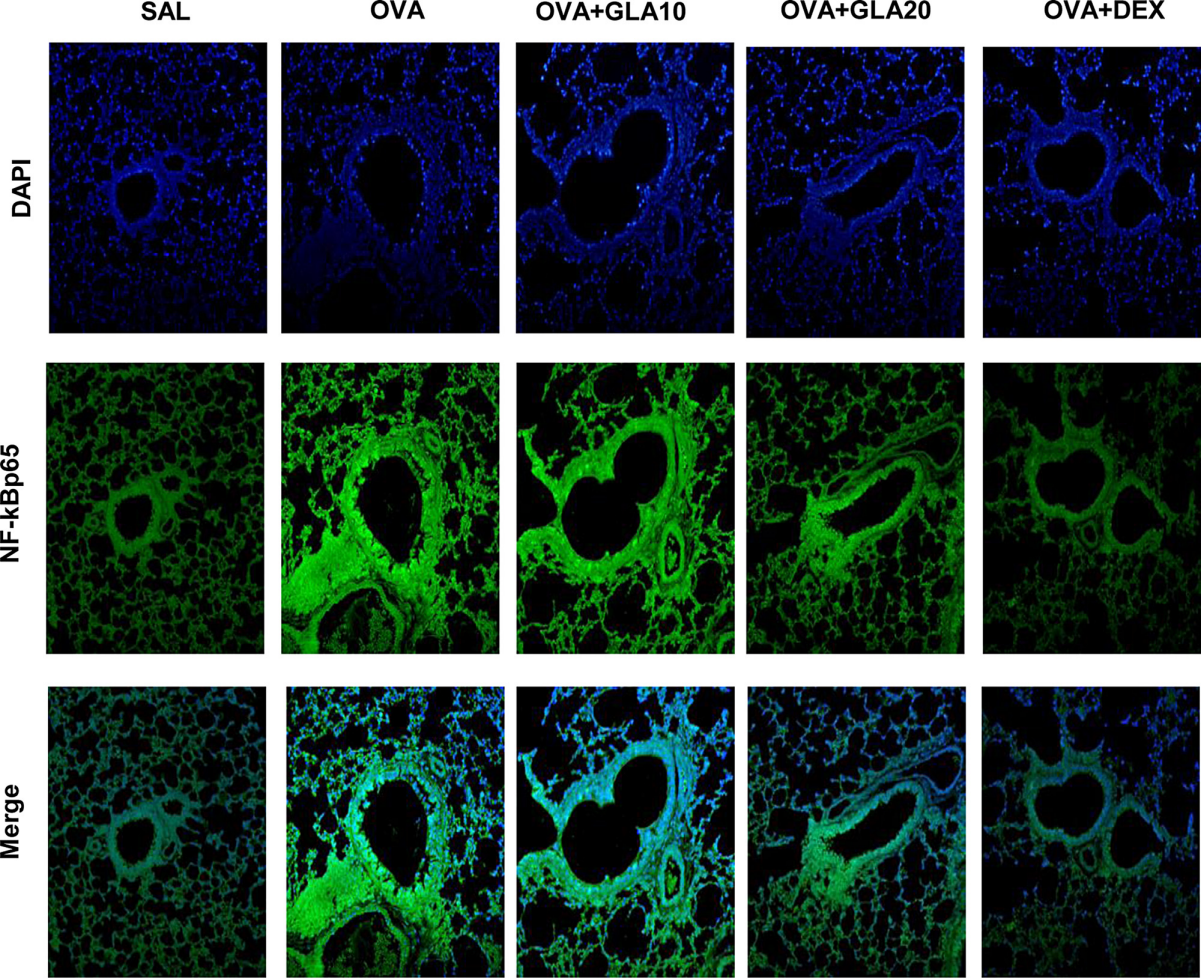
Effects of GLA on nuclear transcription of NF-κBp65 in lung tissue. The nuclear transcription of NF-κBp65 in lung tissue in the SAL, OVA-sensitized, Dex (1 mg/kg), and 10 and 20 mg/kg GLA groups was detected with immunofluorescence (200×). The fluorescent-specific antibody (green) stained NF-κBp65.

To investigate the effects of GLA on NF-κBp65 expression levels in the lung tissue, immunofluorescence was performed. As shown in Figure 9, our results showed that compared with the SAL group, the expression level of NF-κBp65 was significantly increased in the OVA-sensitized group. Moreover, after the treatments of GLA, the expression level of NF-κBp65 was decreased in a dose-dependent manner compared with the OVA-sensitized group. These results suggest that GLA can significantly inhibit the expression level of NF-κBp65 in the lung tissue.

### 3.8 Effects of GLA on inflammatory mediators and cytokines in LPS-stimulated RAW264.7 cells

**Figure 10:**
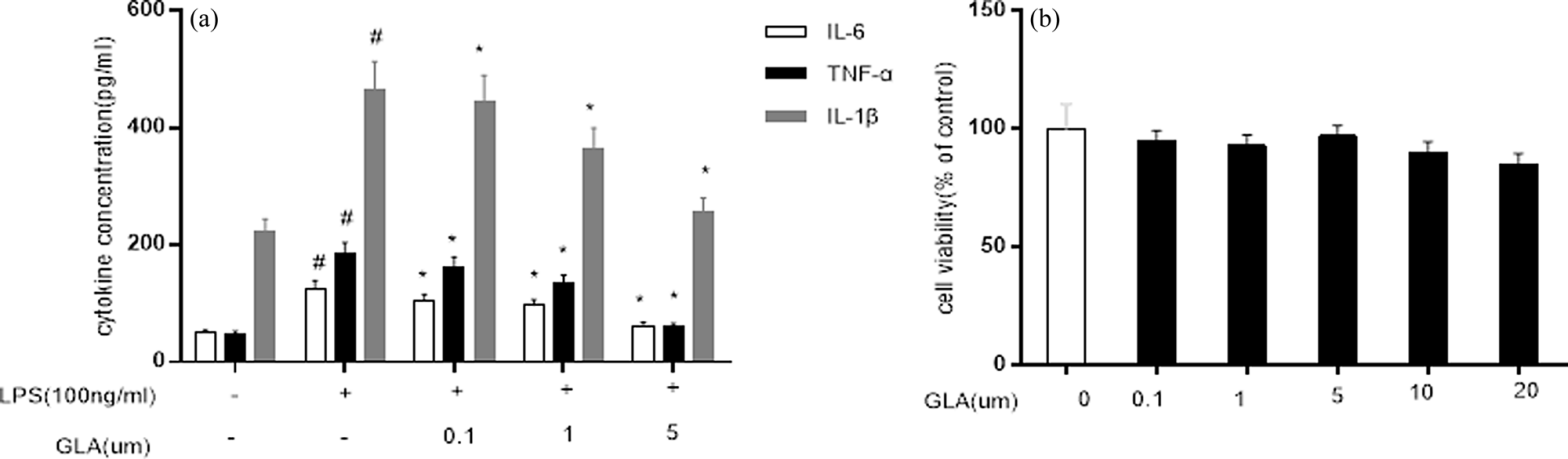
Effects of GLA on pro-inflammatory cytokine production and cellular viability. (a) Production of TNF-α, IL-6, and IL-1β in the LPS-stimulated RAW264.7 cells, in the presence or absence of GLA (at indicated concentrations) was detected with ELISA. (b) Cell viability of the LPS-induced macrophage RAW264.7, treated with GLA (at indicated concentrations), was detected with the MTT assay. Compared with the SAL group, ^#^ *P* < 0.05; and compared with the LPS-treated group, ^*^ *P* < 0.05. *N* = 3; analyzed by ANOVA.

It has been shown that macrophages play important roles in the development of allergic asthma. Therefore, the effects of GLA on production of pro-inflammatory cytokines, such as TNF-α, IL-6, and IL-1β, were investigated, in the RAW264.7 cells treated with 100 ng/mL LPS, in the presence or absence of GLA (0.1, 1, and 5 μM, respectively). As shown in Figure 10a, the expression levels of TNF-α, IL-6, and IL-1β were significantly raised at 6 h after the treatment of LPS, which were reversed by the pretreatment of GLA. These results suggest that GLA can inhibit the release of inflammatory mediators in LPS-induced RAW264.7 cells. In addition, as shown in Figure 10b, the macrophages were treated with LPS in the presence of 5 μM GLA, exhibiting the highest cell survival rate.

### 3.9 Effects of GLA on LPS-induced NF-κB nucleus translocation

**Figure 11:**
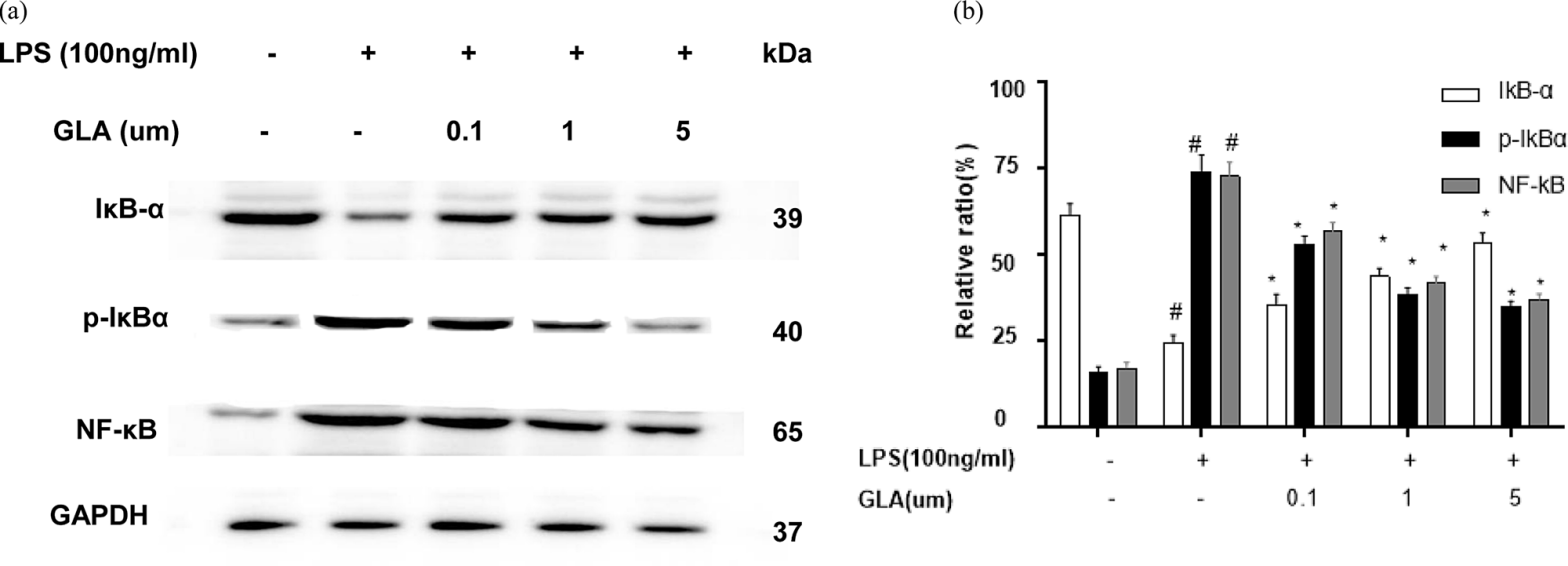
Effects of GLA on the expression of IκB-α, p-IκBα, and NF-κBp65. (a) Expression levels of IκB-α, p-IκBα, and NF-κBp65 in the LPS-stimulated RAW264.7 cells, in the presence or absence of GLA (at indicated concentrations), were detected with Western blot analysis. (b) Statistical analysis of the expression levels of IκB-α, p-IκBα, and NF-κBp65. Compared with the SAL group, ^#^ *P* < 0.05; and compared with the LPS-treated group, ^*^ *P* < 0.05. *N* = 3; analyzed by ANOVA.

Activation of NF-κB depends on the phosphorylation and degradation of IκB-α. Therefore, the expression levels of IκB-α, pIκB-α, and NF-κB were detected by Western blot analysis. Our results showed that the expression levels of pIκB-α and NF-κB were increased, while the IκB-α expression level was decreased in the LPS-stimulated RAW264.7 cell culture medium. After the GLA treatment at indicated concentrations (0, 0.1, 1, and 5 μM), the expression levels of pIκB-α and NF-κB were decreased, while the IκB-α expression was increased (Figure 11), with statistical significance for the GLA (5 μM) group. These results suggest that GLA can block the activation of NF-κB by inhibiting the phosphorylation and degradation of IκB-α.

### 3.10 Effects of GLA on LPS-induced level of NF-κB expression

**Figure 12:**
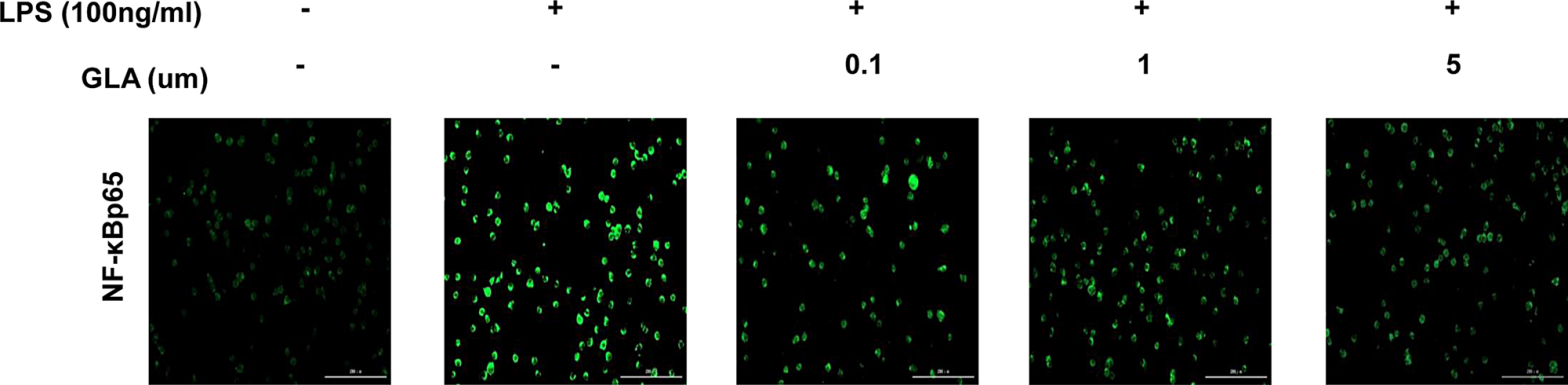
Effects of GLA on NF-κBp65 nuclear translocation in RAW264.7 cells. The expression level of NF-κBp65 in the LPS-stimulated RAW264.7 cells, in the presence or absence of GLA (at indicated concentrations), was detected with immunofluorescence (200×). The experiments were repeated at three times.

In order to further verify whether GLA inhibited the level of NF-κBp65 by immunofluorescence. As shown in Figure 12, in the LPS group, NF-κBp65 expression was upregulated significantly and, however, was downregulated after the GLA treatment dose dependently. These results suggest that GLA can inhibit the NF-κBp65 expression when challenged with LPS on RAW264.7 cells.

## 4 Discussion

In this study, the effects of GLA on the inflammatory responses *in vivo* and *in vitro* asthmatic models were investigated, and the possibly underlying mechanisms concerning the TLR4/NF-κB signaling pathway were analyzed. Our results showed that GLA could inhibit the airway inflammation of OVA-induced asthmatic mice, including alleviation of airway hyperreactivity, reduction of inflammatory cells recruitment, and inhibition of the mucus plug formation and goblet cell proliferation around the airway. In addition, GLA reduced the expression levels of IL-4, IL-5, IL-13, TNF-α, IL-6, and IL-1β and increased the production of IL-12 and IFN-γ. GLA could also downregulate the expression of TLR4 in the lung of asthma mice. GLA blocked the TLR4/NF-κB signaling pathway, consisting of TLR4, MyD88, TRAF6, as well as IκB-α phosphorylation and NF-κB nuclear translocation. In the *in vitro* settings, GLA reduced TNF-α, IL-6, and IL-1β expressions after LPS challenge and the NF-κB expression and nuclear translocation. These results suggest that GLA inhibits the inflammatory responses in asthmatic mice by blocking the TLR4/NF-κB signaling pathway.

T lymphocytes are divided into the CD4^+^ and CD8^+^ cells according to the cell surface antigens. It is confirmed that the CD4^+^ cells can develop into the Th1 and Th2 sub-groups, which can secrete a variety of cytokines and play immunomodulatory roles in the pathogenesis of asthma. IL-12 and IFN-γ are important cytokines of Th1 immune response, while the IL-4, IL-5, and IL-13 are important cytokines of Th2 immune response. Cytokine dysfunction is a key factor in the asthma attack, i.e., the Th1/Th2 disorder. When the Th2 expression is hyperactive, the IL-4 and IL-13 synthesize specific allergen IgE, which binds to the eosinophil/mast cell membrane high-affinity substance and induces the release of inflammatory waterfall that causes asthma [23]. On the other hand, IL-12 can indirectly inhibit the secretion of IL-4 and IL-13, prevent Th0 from differentiating into Th2, inhibit the immune effect of Th2 subpopulation, reduce the IgE synthesis, and decline the airway resistance and inflammatory infiltration, thereby improving the lung function. Hershey et al. [24] have shown that IL-4 can cause a large amount of eosinophil infiltration in the trachea, and the IL-4 gene mutation is associated with atopic constitution and high IgE syndrome. Moreover, Ruffilli and Bonini [25] have found that the *IL-4*, *IL-5*, and *IL-13* genes are located in the 5q31–33 region, which are related to the serological total IgE levels. In China, the factors causing asthma are closely related to the gene expression of IL-12 in the body. IL-12 is a proinflammatory cytokine, always secreted in the form of heterodimers, which can induce the production of IFN-γ, promote the transition of Th0 cells into Th1 cells, and participate in the *in vivo* immune responses. Chen et al. [26] have shown that people without IL-12 are less likely to develop asthma compared to those with IL-12. On the other hand, Kodama et al. [27] have shown that IL-12 can increase the degranulation by eosinophil, thus improving the asthma incidence. GLA is a representative of diterpenoid compounds extracted from cymbidium calyx. It is shown that GLA has anti-inflammatory, anti-tumor, and immunomodulatory effects [28]. Hou et al. [29] demonstrated that GLA could alleviate LPS-induced septic shock and inflammation via inhibiting NLRP3 inflammasome activation. GLA dose-dependently inhibited the production of IL-1β after LPS stimulation *in vivo*. Piao et al. [18] found that GLA significantly reduced the production of cytokines, including IL-4, TNF-α, IL-1β, IL-13, and IL-8, in the rat basophilic leukemia cells, and peritoneal mast cells *in vitro*. Consistently, our results showed that GLA reversed the pathological damages of the lung tissues in asthmatic mice, reduced the expression levels of IL-4, IL-5, IL-13, TNF-α, IL-6, and IL-1β, and increased the production of IL-12 and IFN-γ. These results suggest that GLA may inhibit airway inflammation in asthmatic mice by inhibiting the inflammatory cytokine production.

**Figure 13:**
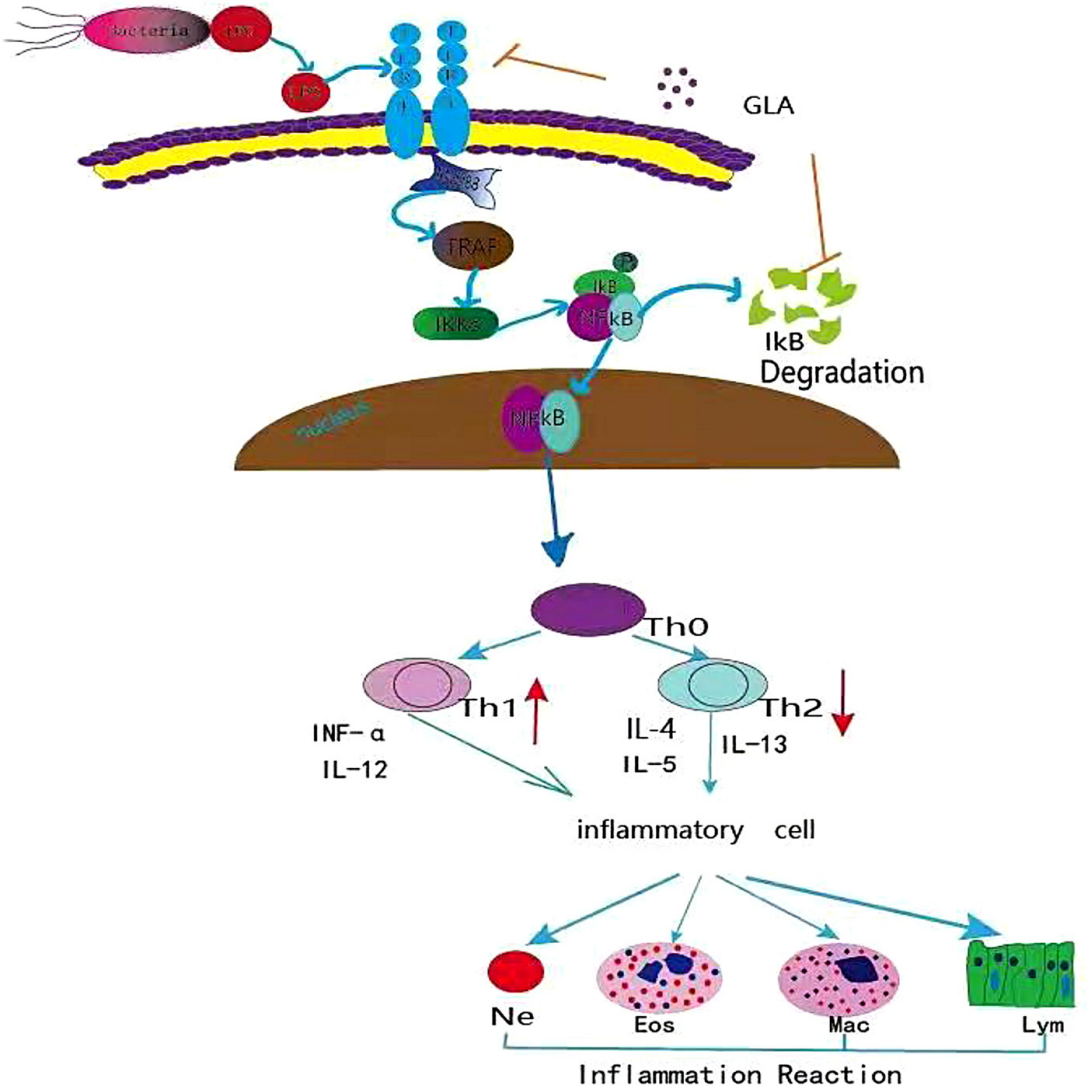
Schematic diagram of the role and mechanism of GLA in asthmatic inflammation. GLA could inhibit the airway inflammatory of asthmatic mice. This effect might be through the downregulation of the expression of TLR4/NF-κB signaling pathways; the inhibition of IL-4, IL-5, IL-13, TNF-α, IL-6, and IL-1β; and the enhancement of IL-12 and IFN-γ levels, as well as inflammatory response mediated by neutrophils, eosinophils, macrophages, and lymphocytes.

TLRs represent the body’s innate immune recognition receptors. When microorganisms break through the body barrier, TLR4 can immediately recognize the event and activate the body to regulate the cellular immune responses [30,31]. LPS, as an activator of TLR4, is a potent inflammation-inducing molecule. LPS is expressed in the antigen-presenting cells by activating the MyD88/NF-κB signal transduction, which promotes the production of intracellular inflammatory transcription factors and mediates the occurrence and development of inflammatory damages. For example, Saturnino et al. [32] have shown that when asthma attack occurs, the spasm of airway smooth muscle can aggravate the airway inflammation and infection. Moreover, TLR4 induces the expression of MyD88 and TRAF6 and promotes the secretion of pro-inflammatory factors (such as IL-4, IL-5, and IL-13), resulting in the airway inflammation and increasing the mucus [32]. The TLR4/NF-κB signaling pathway mediates the inflammatory responses. NF-κB is one of the important factors involved in the pathogenesis of asthma, which has been recognized as a therapeutic target. Luo et al. [33] have shown the elevated expression of TRAF6 in the peripheral blood of allergic rhinitis. Moreover, Riba et al. [34] have shown that TRAF6 may be associated with bronchial asthma. High expression of TRAF6 would activate the NF-κB pathway, which ultimately leads to the chronic inflammation and asthma. Kim et al. [20] have shown that GLA inhibits the activation of NF-κB and degradation of IκBα in LPS-induced microglia and inhibits the production of proinflammatory cytokines TNF-α, IL-1β, and IL-6 to play an anti-neuroinflammatory role. Yang et al. [19] suggested that GLA could alleviate the infiltration of macrophages and neutrophils in lungs and inhibited the activation of NF-κB in fibrotic lungs induced by bleomycin. Chen et al. [26] revealed that GLA treatment could inhibit the phosphorylation of NF-κBp65 and the nuclear expression *in vivo* and *in vitro* in the melanoma cells. Based on the above, we detected the related proteins of TLR4/NF-κB signaling pathway. We found that after treatments of GLA at appropriate concentrations, the expression of TLR4 around the bronchi was significantly decreased, which was least for the high-dose GLA group. Moreover, our results showed that expression levels of NF-κB were inhibited by the GLA treatment in the lung tissue. This was validated by *in vitro* experiments in RAW264.7 macrophages. In summary (Figure 13), the antigens such as endotoxin secreted by bacteria would act on the TLR4 receptor on the cellular surface. MyD88 exerts the biological effects through binding to TLR4, activating TRAF6, and promoting the activation of NF-κB and its translocation into the nucleus. GLA could regulate the Th1/Th2 balance; increase the IL-12 level; decrease the IL-4, IL-5, and IL-13 levels; and inhibit the inflammatory responses in asthma. Therefore, GLA may protect the airway by blocking the TLR4/NF-κB signaling pathway.

## 4 Conclusion

In conclusion, our results showed that GLA inhibited the waterfall-inflammatory responses in the asthmatic mice. GLA downregulated the expression of related proteins possibly by blocking the activation of the TLR4/NF-κB signaling pathway. In view of the immunomodulatory effects, the application of GLA in treating asthma deserves further study. Based on these findings, GLA should be further evaluated as an alternative of targeted drugs for the clinical treatment of asthma.

## References

[1] Song WJ, Kang MG, Chang YS, Cho SH. Epidemiology of adult asthma in Asia: toward a better understanding. Asia Pac Allergy. 2014;4(2):75–85. 10.5415/apallergy.2014.4.2.75 PMC4005350 24809012

[2] Stelmaszczyk-Emmel A. Regulatory T cells in children with allergy and asthma: it is time to act. Respir Physiol Neurobiol. 2015;209:59–63. 10.1016/j.resp.2014.11.010 25462834

[3] Ji NF, Xie YC, Zhang MS, Zhao X, Cheng H, Wang H, et al. Ligustrazine corrects Th1/Th2 and Treg/Th17 imbalance in a mouse asthma model. Int Immunopharmacol. 2014;21(1):76–81. 10.1016/j.intimp.2014.04.015 24785327

[4] Schuijs MJ, Willart MA, Hammad H, Lambrecht BN. Cytokine targets in airway inflammation. Curr Opin Pharmacol. 2013;13(3):351–61. 10.1016/j.coph.2013.03.013 23643194

[5] Han Y, Ye A, Bi L, Wu J, Yu K, Zhang S. Th17 cells and interleukin-17 increase with poor prognosis in patients with acute myeloid leukemia. Cancer Sci. 2014;105(8):933–42. 10.1111/cas.12459 PMC4317867 24890519

[6] Walsh CJ, Zaihra T, Benedetti A, Fugère C, Olivenstein R, Lemière C, et al. Exacerbation risk in severe asthma is stratified by inflammatory phenotype using longitudinal measures of sputum eosinophils. Clin Exp Allergy. 2016;46(10):1291–302. 10.1111/cea.12762 27214328

[7] Ma X, Becker Buscaglia LE, Barker JR, Li Y. MicroRNAs in NF-kappaB signaling. J Mol Cell Biol. 2011;3(3):159–66. 10.1093/jmcb/mjr007 PMC3104013 21502305

[8] Tang Y, Huang W, Song Q, Zheng X, He R, Liu J. Paeonol ameliorates ovalbumin-Induced asthma through the inhibition of TLR4/NF-kappaB and MAPK signaling. Evid Based Complement Altern Med. 2018;2018:3063145. 10.1155/2018/3063145 PMC6114069 30186353

[9] Janssen-Heininger YM, Poynter ME, Aesif SW, Pantano C, Ather JL, Reynaert NL, et al. Nuclear factor kappaB, airway epithelium, and asthma: avenues for redox control. Proc Am Thorac Soc. 2009;6(3):249–55. 10.1513/pats.200806-054RM PMC2677399 19387025

[10] Brady G, Haas DA, Farrell PJ, Pichlmair A, Bowie AG. Molluscum contagiosum virus protein MC005 inhibits NF-kappaB activation by targeting NEMO-regulated IkappaB kinase activation. J Virol. 2017;91(15):e00545–17. 10.1128/JVI.00545-17 PMC5512260 28490597

[11] Durand JK, Baldwin AS. Targeting IKK and NF-kappaB for Therapy. Adv Protein Chem Struct Biol. 2017;107:77–115. 10.1016/bs.apcsb.2016.11.006 28215229

[12] Crespo-Lessmann A, Mateus E, Vidal S, Ramos-Barbón D, Torrejón M, Giner J, et al. Expression of toll-like receptors 2 and 4 in subjects with asthma by total serum IgE level. Respir Res. 2016;17:41. 10.1186/s12931-016-0355-2 PMC4833957 27084682

[13] Gaddis DE, Michalek SM, Katz J. TLR4 signaling via MyD88 and TRIF differentially shape the CD4 + T cell response to Porphyromonas gingivalis hemagglutinin B. J Immunol. 2011;186(10):5772–83. 10.4049/jimmunol.1003192 PMC3809913 21498664

[14] Brickey WJ, Neuringer IP, Walton W, Hua X, Wang EY, Jha S, et al. MyD88 provides a protective role in long-term radiation-induced lung injury. Int J Radiat Biol. 2012;88(4):335–47. 10.3109/09553002.2012.652723 PMC3629725 22248128

[15] Yang J, Liu Y, Xue C, Yu W, Zhang J, Qiao C. Synthesis and biological evaluation of glaucocalyxin A derivatives as potential anticancer agents. Eur J Med Chem. 2014;86:235–41. 10.1016/j.ejmech.2014.08.061 25164762

[16] Ren L, Jing J, Chen G, Miao Y, Wei P. Preparation, characteristic and pharmacological study on inclusion complex of sulfobutylether-β-cyclodextrin with glaucocalyxin A. J Pharm Pharmacol. 2014;66(7):927–34. 10.1111/jphp.12219 24697809

[17] Xiang Z, Wu X, Liu X, Jin Y. Glaucocalyxin A: a review. Nat Prod Res. 2014;28(24):2221–36. 10.1080/14786419.2014.934235 25033290

[18] Piao Y, Jiang J, Wang Z, Wang C, Jin S, Li L, et al. Glaucocalyxin A Attenuates Allergic Responses by Inhibiting Mast Cell Degranulation through p38MAPK/NrF2/HO-1 and HMGB1/TLR4/NF-κB Signaling Pathways. Evid Based Complement Altern Med. 2021;2021:6644751. 10.1155/2021/6644751 PMC8110394 34007295

[19] Yang F, Cao Y, Zhang J, You T, Zhu L. Glaucocalyxin A improves survival in bleomycin-induced pulmonary fibrosis in mice. Biochem Biophys Res Commun. 2017;482(1):147–53. 10.1016/j.bbrc.2016.11.003 27816453

[20] Kim BW, Koppula S, Hong SS, Jeon SB, Kwon JH, Hwang BY, et al. Regulation of microglia activity by glaucocalyxin-A: attenuation of lipopolysaccharide-stimulated neuroinflammation through NF-κB and p38 MAPK signaling pathways. PLoS One. 2013;8(2):e55792. 10.1371/journal.pone.0055792 PMC3564949 23393601

[21] Fulkerson PC, Fischetti CA, Hassman LM, Nikolaidis NM, Rothenberg ME. Persistent effects induced by IL-13 in the lung. Am J Respir Cell Mol Biol. 2006;35(3):337–46. 10.1165/rcmb.2005-0474OC PMC2643287 16645178

[22] Wang C, Choi YH, Xian Z, Zheng M, Piao H, Yan G. Aloperine suppresses allergic airway inflammation through NF-kappaB, MAPK, and Nrf2/HO-1 signaling pathways in mice. Int Immunopharmacol. 2018;65:571–9. 10.1016/j.intimp.2018.11.003 30415164

[23] Choi YH, Yan GH. Silibinin attenuates mast cell-mediated anaphylaxis-like reactions. Biol Pharm Bull. 2009;32(5):868–75. 10.1248/bpb.32.868 19420756

[24] Hershey GK, Friedrich MF, Esswein LA, Thomas ML, Chatila TA. The association of atopy with a gain-of-function mutation in the alpha subunit of the interleukin-4 receptor. N Engl J Med. 1997;337(24):1720–5. 10.1056/NEJM199712113372403 9392697

[25] Ruffilli A, Bonini S. Susceptibility genes for allergy and asthma. Allergy. 1997;52(3):256–73. 10.1111/j.1398-9995.1997.tb00990.x 9140516

[26] Chen T, Liang W, Gao L, Wang Y, Liu Y, Zhang L, et al. Association of single nucleotide polymorphisms in interleukin 12 (IL-12A and -B) with asthma in a Chinese population. Hum Immunol. 2011;72(7):603–6. 10.1016/j.humimm.2011.03.018 21513752

[27] Kodama T, Kuribayashi K, Nakamura H, Fujita M, Fujita T, Takeda K, et al. Role of interleukin-12 in the regulation of CD4 + T cell apoptosis in a mouse model of asthma. Clin Exp Immunol. 2003;131(2):199–205. 10.1046/j.1365-2249.2003.02073.x PMC1808630 12562378

[28] Han M, Li Z, Guo Y, Zhang J, Wang X. A nanoparticulate drug-delivery system for glaucocalyxin A: formulation, characterization, increased in vitro, and vivo antitumor activity. Drug Deliv. 2016;23(7):2457–63. 10.3109/10717544.2015.1012311 25715810

[29] Hou X, Xu G, Wang Z, Zhan X, Li H, Li R, et al. Glaucocalyxin A alleviates LPS-mediated septic shock and inflammation via inhibiting NLRP3 inflammasome activation. Int Immunopharmacol. 2020;81:106271. 10.1016/j.intimp.2020.106271 32062071

[30] Gao D, Li W. Structures and recognition modes of toll-like receptors. Proteins. 2017;85(1):3–9. 10.1002/prot.25179 27699870

[31] Lemaitre B, Nicolas E, Michaut L, Reichhart JM, Hoffmann JA. Pillars article: the dorsoventral regulatory gene cassette spatzle/Toll/cactus controls the potent antifungal response in Drosophila adults. Cell. 1996;86:973–83; J Immunol. 2012;188(11):5210–20. 22611248

[32] Saturnino SF, Prado RO, Cunha-Melo JR, Andrade MV. Endotoxin tolerance and cross-tolerance in mast cells involves TLR4, TLR2 and FcepsilonR1 interactions and SOCS expression: perspectives on immunomodulation in infectious and allergic diseases. BMC Infect Dis. 2010;10:240. 10.1186/1471-2334-10-240 PMC2930646 20707930

[33] Luo X, Hong H, Tang J, Wu X, Lin Z, Ma R, et al. Increased expression of miR-146a in children with allergic rhinitis after allergen-specific immunotherapy. Allergy Asthma Immunol Res. 2016;8(2):132–40. 10.4168/aair.2016.8.2.132 PMC4713876 26739406

[34] Riba M, Garcia Manteiga JM, Bošnjak B, Cittaro D, Mikolka P, Le C, et al. Revealing the acute asthma ignorome: characterization and validation of uninvestigated gene networks. Sci Rep. 2016;6:24647. 10.1038/srep24647 PMC4838989 27097888

